# The International Conference on Intelligent Biology and Medicine (ICIBM) 2018: systems biology on diverse data types

**DOI:** 10.1186/s12918-018-0648-9

**Published:** 2018-12-21

**Authors:** Kai Wang, Xiaoming Liu, Yan Guo, Zhijin Wu, Degui Zhi, Jianhua Ruan, Zhongming Zhao

**Affiliations:** 1 Raymond G. Perelman Center for Cellular and Molecular Therapeutics, Children’s Hospital of Philadelphia, Philadelphia, PA 19104 USA; 2 Department of Pathology and Laboratory Medicine, University of Pennsylvania, Philadelphia, PA 19104 USA; 3 Human Genetics Center, School of Public Health, The University of Texas Health Science Center at Houston, Houston, TX 77030 USA; 4 College of Public Health, University of South Florida, Tampa, FL 33612 USA; 5 Comprehensive Cancer Center, University of New Mexico, Albuquerque, NM 87131 USA; 6 Department of Biostatistics, Brown University, Providence, RI 02912 USA; 7 Center for Precision Health, School of Biomedical Informatics, The University of Texas Health Science Center at Houston, Houston, TX 77030 USA; 8 Department of Computer Science, The University of Texas at San Antonio, San Antonio, TX 78249 USA

## Abstract

Between June 10–12, 2018, the International Conference on Intelligent Biology and Medicine (ICIBM 2018) was held in Los Angeles, California, USA. The conference included 11 scientific sessions, four tutorials, one poster session, four keynote talks and four eminent scholar talks that covered a wide range of topics in 3D genome structure analysis and visualization, next generation sequencing analysis, computational drug discovery, medical informatics, cancer genomics and systems biology. Systems biology has been a main theme in ICIBM 2018, with exciting advances presented in many areas of systems biology, covering various different data types such as gene regulation, circular RNAs expression, single-cell RNA-Seq, inter-chromosomal interactions, metabolomics, proteomics and phosphoproteomics. Here, we describe ten high quality papers to be published in BMC Systems Biology.

## Introduction

For the past six years, ICIBM meeting has been covering extensive cutting edge research topics in systems biology. There is no exception to ICIBM 2018. As the systems biology research advances, research focus has been shifted from methodology development to the application of systems biology approach to solve practical biomedical problems. We selected ten high quality papers from ICIBM 2018 meeting to reflect this trend, covering a range of topics in systems biology. Traditional topics in systems biology, such as gene regulatory network inference and gene signature/set analysis, are covered in this supplement issue. Additionally, systems biology analysis of additional data types, including circular RNAs, single-cell RNA-Seq, inter-chromosomal interactions, metabolomics, proteomics and phosphoproteomics, are also represented in this supplement issue. Altogether, these papers present various cutting edge researches in systems biology, suggesting that as a field, systems biology is playing increasingly important roles in biology and medicine, to infer novel biological insights from large-scale data sets and potentially facilitate clinical applications. Below, we briefly summarize the main findings from each paper.

## The science program for the ICIBM 2018 systems biology track

In the first paper by Dehghannasiri et al. [1], the authors proposed an experimental design framework for Markovian gene regulatory networks under stationary control policy. The design framework is based on the concept of the mean objective cost of uncertainty (MOCU), which quantifies uncertainty based on the performance degradation caused by uncertainty. The unknown parameters are governed by a prior distribution, which can be updated to a posterior distribution by observing a regulatory trajectory. They selected an experiment that minimizes the MOCU remaining after incorporating its output into the network model. To mitigate the computational complexity, they also implemented an approximate experimental design method utilizing mean first passage times (MFPTs). Analysis on synthetic and real gene regulatory networks demonstrate the efficacy of the proposed method, including the accuracy and computational advantage of the approximate MFPT-based design.

Gene signatures are important to represent molecular changes in disease genomes, and are useful to separate samples into different groups for clinical treatment. In the second paper by Mallik et al. [2], the authors proposed a new framework for identifying gene signatures using Pareto-optimal cluster size for RNA-Seq data. They conducted Limma analysis to identify differentially expressed genes (DEGs). They next applied k-means clustering using optimal cluster size obtained by Multi-objective Optimization for Collecting Cluster Alternatives (MOCCA) technique. For The Cancer Genome Atlas (TCGA) cervical cancer RNA-Seq dataset, they identified a multi-objective gene signature (best cluster having highest average correlation) containing 35 DEGs that could classify cancer samples with higher classification accuracy (0.935 using PAMR classifier). Their method may be useful to find signature from any RNA-Seq or microarray data.

The third paper by Yang et al. [3] focused on systems biology of gene expression on a different type of transcripts, circular RNAs (circRNAs). There have been no reports of circRNAs expression profiles during the differentiation of mouse neural stem cells (NSCs) and the authors tried to find the possible regulatory roles of circRNAs in the differentiation. In this study, they first sequenced total RNA from NSCs from the fetal mouse cerebral cortex. By analyzing the RNA-Seq data, they found 37 circRNAs and 4182 mRNAs that were differentially expressed during the NSC differentiation. Then, they performed a co-expression network analysis of these differentially expressed circRNAs and mRNAs. The result suggested a stronger Gene Ontology (GO) enrichment in neural features for both the cognate linear genes of circRNAs and differentially expressed mRNAs. The network analysis in the study suggested the possible complex circRNA-mRNA mechanisms during NSC differentiation.

Gene superset, which is an unbiased combination of gene sets, is the topic of the next paper. In the next study by Chen et al. [4], the authors proposed a gene superset autoencoder (GSAE), a multi-layer autoencoder model with the incorporation of a priori defined gene sets that retain the crucial biological features in the latent layer. They introduced the concept of the gene superset, an unbiased combination of gene sets with weights trained by the autoencoder, where each node in the latent layer is a superset. They demonstrated that gene supersets could retain sufficient biological information with respect to tumor subtypes and clinical prognostic significance. The superset also provides high reproducibility on survival analysis and accurate prediction for cancer subtypes.

Single-cell RNA sequencing (scRNA-Seq) is an emerging technology that has revolutionized the research of the tumor heterogeneity and other biomedical fields. However, due to data sparsity, the study of differential gene regulatory networks using scRNA-Seq data remains a challenging task. In the next paper by Chiu et al. [5], the authors proposed and implemented a bioinformatics tool, namely scdNet, for sample size adjustment of gene-gene correlation, comparison of inter-state correlations, and construction of differential networks using scRNA-Seq data. Performance of scdNet has been verified by simulated and real datasets, including two datasets of single circulating tumor cells (CTCs) of prostate cancer and early mouse embryos. The tool may be widely applicable to future scRNA-Seq datasets and infer novel biological insights.

The next paper from Dai et al. [6] focuses on inter-chromosomal interactions. Unlike metazoan where CCCTC-binding factor (CTCF) plays major roles in mediating chromatin interactions, in yeast, the transcription factors (TFs) involved in this biological process are poorly known. Under the rationale that TFs enriched in the strong inter-chromosomal interactions are more likely to play major roles in mediating inter-chromatin interactions, the authors presented two computational approaches (Chi-square method and multivariate linear regression) to estimate the TFs enriched in the chromatin physical inter-chromosomal interactions in yeast. They found 10 enriched TFs using both computational approaches. Among them, they highlighted two significantly enriched TFs, Ste12 and Dig1, which had been reported to be involved in centromeric transcript maintaining and spatial organization. No TF in this study was found to have a dominant impact on the inter-chromosomal interaction as CTCF did in the human or other metazoan, suggesting that species without CTCF might have a different system in mediating inter-chromosomal interactions. In summary, they presented a systematic examination of TFs involved in chromatin interaction in yeast and provided candidate TFs for future studies.

The next paper by Choi et al. [7] focuses on the topic of metabolomics. The mammalian brain is organized into regions with specific properties and biological functions. These regions have distinct transcriptomes, but little is known whether they may also differ in their metabolome. To examine the metabolome of various brain regions, the authors used mass spectrometry and several analytical algorithms. All four brain regions in this study had a unique metabolic signature, but the associated metabolites came from all chemical categories and were not pathway-centric. These data thus indicated the diversity of global brain metabolome corresponding to specialized regional brain functions and provided a new perspective on the underlying properties of brain regions.

The next study from San Lucas et al. [8] described a network-based analysis on gene expression profiles in order to identify functional gene subnetworks involved in mild traumatic brain injury (TBI). TBI represents a critical health problem of which timely diagnosis and treatment currently remain challenging. The gene expression profiles were obtained from two experimental models of injury in rats: the controlled cortical impact and the fluid percussion injury. The method integrates protein interaction information with gene expression profiles to identify subnetworks of genes as biomarkers. The authors have demonstrated that the identified gene subnetworks are more accurate to classify the heterogeneous responses to different injury models, compared to conventional analysis using individual marker genes selected without network information. Therefore, the systems approach leads to a better understanding of the underlying complexities of the molecular responses after TBI and the identified subnetworks will have important prognostic functions for patients who sustain mild TBIs.

The next study by Ren et al. [9] integrated proteomic and phosphoproteomic data to perform pathway prioritization in breast cancer. Three different strategies including Hypergeometric test based over-representation analysis, Kolmogorov-Smirnov test based gene set analysis and topology-based pathway analysis, were applied and evaluated in integrating protein expression and phosphorylation. In comparison, the authors also assessed the ranking performance of the strategy using information of protein expression or protein phosphorylation individually. The results demonstrate that integrative strategy performs best and the network topology-based method is more powerful by integrating proteomic and phosphoproteomic in pathway analysis of proteomics study. They also applied topology-based pathway analysis with integrating protein expression and phosphorylation profiles on four subtypes of breast cancer. Significantly different results were showed among the subtypes, and were consistent with some previous researches. The results demonstrate that the network topology-based method is more powerful by integrating proteomic and phosphoproteomic in pathway analysis of proteomics study.

The last paper by Wang et al. [10] in this supplement issue focuses on proteins. In the study, the authors proposed a deep learning method for efficient prediction of self-interactions protein (SIP). In particular, they proposed a Stacked Long Short-Term Memory (SLSTM) neural network that contains “dropout”. Experimental results showed that this model could efficiently predict SIP and greatly improve the accuracy of current SIP predictions. Additionally, the authors recognized that the previous methods for predicting the SIPs often use the physicochemical or structural information of the target protein, which is complex and not easily accessible. Instead, they proposed an image processing technique to efficiently extract the evolutionary information of proteins for representing specific proteins. This approach only considers the evolutionary information of proteins, which is easily popularized. This study is among the first to apply deep learning method to predict SIPs, and practical experimental results revealed its potential in SIPs identification.

## References

[1] Dehghannasiri R, Esfahani MS, Dougherty ER. An experimental design framework for Markovian gene regulatory networks under stationary control policy. BMC Syst Biol. 2018;S1. 10.1186/s12918-018-0649-8 10.1186/s12918-018-0649-8 PMC6302376 30577732

[2] Mallik S, Zhao Z. Identification of gene signatures from RNA-seq data using Pareto-optimal cluster algorithm. BMC Syst Biol. 2018;S2. 10.1186/s12918-018-0650-2 10.1186/s12918-018-0650-2 PMC6302366 30577846

[3] Yang Q, Wu J, Zhao J, Xu T, Zhao Z, Song X, Han P. Circular RNA expression profiles during the differentiation of mouse neural stem cells. BMC Syst Biol. 2018;S3. 10.1186/s12918-018-0651-1 10.1186/s12918-018-0651-1 PMC6302452 30577840

[4] Chen H-IH, Chiu Y-C, Zhang T, Zhang S, Huang Y, Chen Y. GSAE: an autoencoder with embedded gene-set nodes for genomics functional characterization. BMC Syst Biol. 2018;S4. 10.1186/s12918-018-0642-2 10.1186/s12918-018-0642-2 PMC6302374 30577835

[5] Chiu Y-C, Hsiao T-H, Wang L-J, Chen Y, Y-HJ S. scdNet: a computational tool for single-cell differential network analysis. BMC Syst Biol. 2018;S5. 10.1186/s12918-018-0652-0 10.1186/s12918-018-0652-0 PMC6302455 30577836

[6] Dai Y, Li C, Pei G, Dong X, Ding G, Zhao Z, Li Y, Jia P. Multiple transcription factors contribute to inter-chromosomal interaction in yeast. BMC Syst Biol. 2018;S6. 10.1186/s12918-018-0643-1 10.1186/s12918-018-0643-1 PMC6302461 30577873

[7] Choi WT, Tosun M, Jeong H-H, Karakas C, Semerci F, Liu Z, Maletić-Savatić M. Metabolomics of mammalian brain reveals regional differences. BMC Syst Biol. 2018;S7. 10.1186/s12918-018-0644-0 10.1186/s12918-018-0644-0 PMC6302375 30577853

[8] San Lucas FA, Redell J, Pramod D, Liu Y. Classifying mild traumatic brain injuries with functional network analysis. BMC Syst Biol. 2018;S8. 10.1186/s12918-018-0645-z 10.1186/s12918-018-0645-z PMC6302365 30577783

[9] Ren J, Wang B, Li J. Integrating proteomic and phosphoproteomic data for pathway analysis in breast cancer. BMC Syst Biol. 2018;S9. 10.1186/s12918-018-0646-y 10.1186/s12918-018-0646-y PMC6302460 30577793

[10] Wang Y-B, You Z-H, Li X, Jiang T-H, Cheng L, Chen Z-H. Prediction of protein self-interactions using stacked long short-term memory from protein sequences information. BMC Syst Biol. 2018;S10. 10.1186/s12918-018-0647-x 10.1186/s12918-018-0647-x PMC6302371 30577794

